# Toward an elucidation of the molecular genetics of inherited retinal degenerations

**DOI:** 10.1093/hmg/ddx185

**Published:** 2017-05-16

**Authors:** G. Jane Farrar, Matthew Carrigan, Adrian Dockery, Sophia Millington-Ward, Arpad Palfi, Naomi Chadderton, Marian Humphries, Anna Sophia Kiang, Paul F. Kenna, Pete Humphries

**Affiliations:** 1 Institute of Genetics, School of Genetics and Microbiology, University of Dublin, Trinity College, Dublin 2, Ireland; 2 Research Foundation, Royal Victoria Eye and Ear Hospital, Dublin 2, Ireland

## Abstract

While individually classed as rare diseases, hereditary retinal degenerations (IRDs) are the major cause of registered visual handicap in the developed world. Given their hereditary nature, some degree of intergenic heterogeneity was expected, with genes segregating in autosomal dominant, recessive, X-linked recessive, and more rarely in digenic or mitochondrial modes. Today, it is recognized that IRDs, as a group, represent one of the most genetically diverse of hereditary conditions - at least 260 genes having been implicated, with 70 genes identified in the most common IRD, retinitis pigmentosa (RP). However, targeted sequencing studies of exons from known IRD genes have resulted in the identification of candidate mutations in only approximately 60% of IRD cases. Given recent advances in the development of gene-based medicines, characterization of IRD patient cohorts for known IRD genes and elucidation of the molecular pathologies of disease in those remaining unresolved cases has become an endeavor of the highest priority. Here, we provide an outline of progress in this area.

## Genetic Heterogeneity in IRDs

Inherited retinal degenerations (IRDs) represent the most frequent cause of visual dysfunction in those of working age, such conditions therefore having a highly significant impact on quality of life and health economics. The more common IRDs include Retinitis Pigmentosa (RP), Choroideremia, Leber congenital amaurosis (LCA), Usher syndrome, Congenital stationary night blindness (CSNB), Vitelliform macular dystrophy, Stargardt Macular Dystrophy, Best disease, Retinoschisis, cone-rod dystrophy and myocilin-based hereditary forms of open angle glaucoma (see www.sph.uth.tmc.edu/retnet/ for details of disorders, causative genes and mutations). Typically, IRDs result in gradual photoreceptor loss and compromised vision, often leading to registered blindness. Patterns of inheritance include autosomal recessive, autosomal dominant and X-linked, however, more rare mitochondrial and digenic forms of retinal dystrophies have been characterized.

Since the localization, through genetic linkage analysis, of the first IRD genes involved in X-linked recessive and autosomal dominant forms of RP (1–3), relentless progress has been made over three decades in defining an extensive portfolio of disease-causing genes, revealing an extreme level of genetic heterogeneity (4,5). Thus far, approximately 260 genes have been characterised, with more still to be identified (6–9; Tables 1–3). DNA sequencing, and more recently, next-generation sequencing (NGS) technologies, in particular, now provide an opportunity to characterize the majority of the remaining genes (10–25).

**Table 1:** Representative NGS studies of IRD patient populations

IRD patient numbers	Patient origin (predominant)	NGS technology	Pathogenic/likely pathogenic mutations (%)	Reference
100	Dutch	Target capture 111 genes	∼ 50%	Neveling *et al.* 2012 (10)
20	France	Target capture 254 genes	57%	Audo *et al.* 2012 (11)
50	UK	Target capture 105 genes	50-50%	O’Sullivan *et al.* 2012 (12)
50	UK	Target capture 73 genes	53% (early onset IRD;25% overall)	Shanks *et al.* 2013 (13)
192	US	Target capture (GEDi) 214 genes	51%	Consugar *et al.* 2015 (14)
105	eyeGENE® database	Target capture 195 genes	49.5%	Ge *et al.* 2015 (15)
82	N Ireland	Target capture 186 genes	60%	Zhao *et al.* 2015 (16)
88	Italy	Target capture 72 genes	59.1%	Bernardis *et al.* 2016 (17)
309	Ireland	Target capture 216 genes	57%	Carrigan *et al.* 2016 (18)
537	Worldwide	Target capture 105 genes	51%	Ellingford *et al.* 2016 (19)
47	Spain	Target capture 75 genes	57.4%	Perez-Carro *et al.* 2016 (20)
88	European	Target capture & WES	61%	Weisschuh *et al.* 2016 (21)
58	Swiss	WES	64%	Tiwari *et al.* 2016 (22)
33	Mixed	WES	55%	de Castro-Miró *et al.* 2016 (23)
59	Spain	WES	71.8%	Riera *et al.* 2017 (24)
266	Dutch	Target panel & WES	52%	Haer-Wigman *et al.* 2017 (25)

**Table 2:** Recessive Retinitis Pigmentosa genes, localizations and functions

Gene	Location	Function
**ABCA4**	1p22.1	ATP binding cassette subfamily A member 4; Photoreceptor-specific ATP-binding cassette (ABC) transporter with N-retinylidene-PE as a substrate.
**AGBL5**	2p23.3	ATP/GTP binding protein like 5; Metallocarboxypeptidase that mediates protein deglutamylation; can remove glutamate residues from both carboxyl termini and side chains of protein substrates.
**ARL6**	3q11.2	ADP ribosylation factor like GTPase 6; Involved in membrane protein trafficking at the base of the cilium.
**ARL2BP**	16q13.3	ADP ribosylation factor like GTPase 2 binding protein; Binds to ARL2.GTP with high affinity. Role in nuclear translocation, retention and transcriptional activity of STAT3.
**BBS1**	11q13.2	Bardet-Biedl syndrome 1 protein; Required for BBSome complex assembly, its ciliary localization and ciliogenesis.
**BBS2**	16q13	Bardet-Biedl syndrome 2 protein; Required for BBSome complex assembly, its ciliary localization and ciliogenesis.
**BEST1**	11q12.3	Bestrophin 1; Forms calcium-sensitive chloride channels; highly permeable to bicarbonate.
**C2orf71**	2p23.2	Chromosome 2 open reading frame 71; Photoreceptor cilia protein connecting inner and outer segments.
**C8orf37**	8q22.1	Chromosome 8 Open Reading Frame 37; Co-localizes with polyglutamylated tubulin at the base of the primary cilium in RPE cells.
**CERKL**	2q31.3	Ceramide kinase-like protein; has no detectable ceramide-kinase activity; protects cells from apoptosis in oxidative stress.
**CLRN1**	3q25.1	Clarin 1; A role in excitatory ribbon synapse junctions between hair cells and cochlear ganglion cells and possibly in analogous synapses in retina.
**CNGA1**	4p12	Cyclic nucleotide gated channel alpha 1; Rod cGMP-gated channel alpha subunit; Forms cGMP-gated cation channel for depolarization of rod photoreceptors.
**CNGB1**	16q21	Cyclic nucleotide gated channel beta 1; Rod cGMP-gated channel beta subunit; Forms cGMP-gated cation channel for depolarization of rod photoreceptors.
**CRB1**	1q31.3	Crumbs 1; Plays a role in photoreceptor morphogenesis in the retina. May maintain cell polarization and adhesion.
**CYP4V2**	4q35.1	Cytochrome P450 family 4 subfamily V member 2; Involved in fatty acid and steroid metabolism in the eye.
**DHDDS**	1p36.11	Dehydrodolichyl Diphosphate Synthase Subunit; forms the dehydrodolichyl diphosphate synthase (DDS) complex, an essential component of the dolichol monophosphate (Dol-P) biosynthetic machinery
**DHX38**	16q22.2	DEAH-Box Helicase 38; Unknown function; probable ATP-binding RNA helicase involved in pre-mRNA splicing.
**EMC1**	1p36.13	ER membrane protein complex subunit 1; Unknown function.
**EYS**	6q12	Eyes Shut Homolog (Drosophila); An extracellular matrix protein, one of the largest human genes at > 2mb. Possibly involved in stability of photoreceptor ciliary axonemes.
**FAM161A**	2p15	Family with sequence similarity 161 member A; Ciliary protein and a member of the Golgi-centrosomal interactome; involved in development of retinal progenitors.
**GNAT1**	3p21.31	G Protein Subunit Alpha Transducin 1; α subunit of the heterotrimeric G-protein transducing; stimulates the coupling of rhodopsin and cGMP-phoshodiesterase.
**GPR125/ADGRA3**	4p15.2	Adhesion G protein-coupled receptor A3/G protein-coupled receptor 125; Involved in signal trasduction.
**HGSNAT**	8p11.21	Heparan-alpha-glucosaminide N-acetyltransferase; Lysosomal acetyltransferase that catalyzes transmembrane acetylation of the terminal glucosamine residues of heparan sulfate, prior to hydrolysis by α-N-acetylglucosaminidase.
**IDH3B**	20p13	Isocitrate Dehydrogenase 3 (NAD(+)) Beta; catalyzes the oxidation of isocitrate to alpha-ketoglutarate in the citric acid cycle.
**IFT140**	16p13.3	Intraflagellar transport 140; Involved in ciliogenesis, cilia maintenance and retrograde ciliary transport.
**IFT172**	2p23.3	Intraflagellar transport 172; Involved in formation and maintenance of cilia.
**IMPG2**	3q12.3	Interphotoreceptor matrix proteoglycan 2; Involved in organization of interphotoreceptor matrix.
**KIAA1549**	7q34	KIAA1549 protein; Unknown function.
**KIZ**	20p11.23	Kizuna centrosomal protein; Localizes to centrosomes, strengthening and stabilizing the pericentriolar region prior to spindle formation.
**LRAT**	4q32.1	Lecithin retinol acyltransferase; Transfers the acyl group from phosphatidylcholine to all-trans retinol, producing all-trans retinyl esters.
**MAK**	6p24.2	Male germ-cell associated kinase; Unknown function.
**MERTK**	2q13	C-mer proto-oncogene receptor tyrosine kinase; A regulator of rod outer segment fragment phagocytosis.
**MVK**	12q24.11	Mevalonate kinase; Involved in the synthesis of isopentenyl diphosphate from (R)-mevalonate.
**NEK2**	1q32.3	NIMA (never in mitosis gene A)-related kinase 2; Involved in regulation of centrosome disjunction.
**NEUROD1**	2q31.3	Neuronal differentiation protein 1; Acts as a transcriptional activator.
**NR2E3**	15q23	Nuclear receptor subfamily 2 group E3; Transcription factor, that is an activator of rod development and repressor of cone development.
**NRL**	14q11.2	Neural retina leucine zipper; Transcription factor which regulates expression of rod-specific genes, involved in photoreceptor cell development and function
**OFD1**	Xp22.2	Oral-facial-digital syndrome 1 protein; Involved in biogenesis of the cilium and controlling centriole length during cell division.
**PDE6A**	5q32	Alpha subunit of cGMP phosphodiesterase; involved in visual transduction cycle.
**PDE6B**	4p16.3	Beta subunit of cGMP phosphodiesterase; involved in visual transduction cycle.
**PDE6G**	17q25.3	Gamma subunit of cGMP phosphodiesterase; involved in visual transduction cycle.
**POMGNT1**	1p34.1	Protein O-linked acetylglucosaminyltransferase 1 (beta 1,2-); Participates in O-mannosyl glycosylation.
**PRCD**	17q25.1	Progressive rod-cone degeneration protein; Unknown function.
**PRIM1**	12q13.3	DNA primase small subunit; Polymerase that synthesizes small RNA primers for Okazaki fragments made during discontinuous DNA replication.
**RBP3**	10q11.22	Retinol-binding protein 3 (IRBP); Shuttles 11-cis and all trans retinoids between the retinol isomerase in RPE and visual pigments in photoreceptor cells.
**RGR**	10q23.1	RPE-retinal G protein-coupled receptor; may be involved in regulating recycling of retinal.
**RHO**	3q22.1	Rhodopsin; Light-induced isomerization of 11-cis to all-trans retinal leading to G-protein activation and release of all-trans retinal.
**RLBP1**	15q26.1	Retinaldehyde-binding protein 1; Soluble retinoid carrier essential for function of photoreceptors.
**RP1**	8q11.23-q12.1	Oxygen-regulated protein 1; Involved in organization of photoreceptor outer segments.
**RP1L1**	8p23.1	Retinitis pigmentosa 1-like 1 protein; Required for differentiation of photoreceptors and organization of photoreceptor outer segments.
**RP2**	Xp11.3	Retinitis pigmentosa 2 (X-linked); Involved in trafficking between Golgi and the ciliary membrane.
**RPE65**	1p31.2	Retinoid isomerohydrolase; Involved in production of 11-cis retinal and visual pigment regeneration.
**RPGR**	Xp11.4	X-linked retinitis pigmentosa GTPase regulator; Involved in ciliogenesis
**SAG**	2q37.1	Rod photoreceptor-arrestin; Binds to photoactivated-phosphorylated rhodopsin, thereby apparently preventing the transducin-mediated activation of phosphodiesterase
**SLC7A14**	3q26.2	Solute carrier family 7 member 14; Unknown function.
**SLC24A1**	15q22.31	Solute carrier family 24 (sodium/potassium/calcium exchanger) member 1; Critical component of visual transduction, controlling the calcium concentration of outer segments.
**SPATA7**	14q31.3	Spermatogenesis associated protein 7; Required for recruitment and localization of RPGRIP1 to the photoreceptor cilium.
**TRNT1**	3p26.2	CCA adding tRNA nucleotidyl transferase 1; Adds and repairs CCA sequences necessary for attachment of amino acids to the 3' terminus of tRNA molecules.
**TTC8**	14q31.3	Tetratricopeptide repeat protein 8; Subunit of the BBSome complex required for proper cilia functions.
**TULP1**	6p21.31	Tubby-like protein 1; Required for normal development of photoreceptor synapses.
**USH2A**	1q41	Usherin; Involved in photoreceptor cell maintenance.
**ZNF408**	11p11.2	Zinc finger protein 408; Unknown function.
**ZNF513**	2p23.3	Zinc finger protein 513; Transcriptional regulator involved in retinal development and maintenance.

**Table 3:** Autosomal Dominant Retinitis Pigmentosa genes, localizations & function

Gene	Location	Function
**ARL3**	10q24.32	ADP-ribosylation factor-like GTPase 3; Required for cytokinesis and cilia signaling.
**BEST1**	11q12.3	Bestrophin-1; Forms calcium-sensitive chloride channels.
**CA4**	17q23.1	Carbonic anhydrase IV; Involved in acid overload removal from retina and acid release in the choriocapillaris.
**CRX**	19q13.33	Cone-rod homeobox protein; Transcription factor that binds and transactivates the 5'-TAATC[CA]-3' sequence found upstream of photoreceptor-specific genes, including opsin genes.
**FSCN2**	17q25.3	Fascin-2; Acts as an actin bundling protein.
**GUCA1B**	6p21.1	Guanylyl cyclase-activating protein 2; Stimulates guanylyl cyclase 1 and 2 in response to changing Ca2+ concentrations.
**HK1**	10q22.1	Hexokinase-1; Catalyses D-hexose + ATP to D-hexose 6-phosphate + ADP.
**IMPDH1**	7q32.1	Inosine-5'-monophosphate dehydrogenase 1; Involved in rate-limiting step in de novo synthesis of guanine nucleotides.
**KLHL7**	7p15.3	Kelch-like protein 7; Substrate-specific adapter of a BCR (BTB-CUL3-RBX1) E3 ubiquitin ligase complex which mediates ubiquitination and degradation of substrate proteins.
**NR2E3**	15q23	Nuclear receptor subfamily 2 group E3; Transcriptional factor that is an activator of rod development and repressor of cone development.
**NRL**	14q11.2	Neural retina lucine zipper; Transcription factor which regulates expression of rod-specific genes.
**PRPF3**	1q21.2	Pre-mRNA-processing-splicing factor 3; A small nuclear ribonucleoprotein, required for the assembly of the spliceosome U4/U5/U6 tri-snRNP complex.
**PRPF4**	9q32	Pre-mRNA-processing-splicing factor 4; A small nuclear ribonucleoprotein, required for the assembly of the spliceosome U4/U5/U6 tri-snRNP complex.
**PRPF8**	17p13.3	Pre-mRNA-processing-splicing factor 8; Acts as a scaffold aiding assembly of spliceosomal proteins.
**PRPF31**	19q13.42	Pre-mRNA-processing-splicing factor 31; A small nuclear ribonucleoprotein, required for the assembly of the spliceosome U4/U5/U6 tri-snRNP complex.
**PRPH2**	6p21.1	Peripherin-2; Involved in photoreceptor disk morphogenesis.
**RDH12**	14q24.1	Retinol dehydrogenase; An NADPH-dependent retinal reductase with high activity toward 9-cis and all-trans-retinol.
**RHO**	3q22.1	Rhodopsin; Light-induced isomerization of 11-cis to all-trans retinal leading to G-protein activation and release of all-trans retinal.
**ROM1**	11q12.3	Rod outer segment membrane protein; Essential for disk morphogenesis.
**RP1**	8q11.23	Oxygen-regulated protein 1; Required for the differentiation of photoreceptor cells, Involved in organization of rod and cone photoreceptor outer segments.
**RP9**	7p14.3	Retinitis pigmentosa 9 protein; possibly a target protein for the PIM1 kinase.
**RPE65**	1p31.3	Retinoid isomerohydrolase; Involved in production of 11-cis retinal and visual pigment regeneration.
**SEMA4A**	1q22	Semaphorin-4A; Promotes axon growth cone collapse.
**SNRNP200**	2q11.2	U5 small nuclear ribonucleoprotein 200 kDa helicase; Involved in spliceosome assembly, activation and disassembly.
**SPP2**	2q37.1	Secreted phosphoprotein 2; Unknown function.
**TOPORS**	9p21.1	E3 ubiquitin-protein ligase Topors; Functions as an E3 ubiquitin-protein ligase and as an E3 SUMO1-protein ligase.

IRD genes and encoded proteins are involved in a wide array of functions. It is of note that mutations in essentially all genes encoding components of the visual transduction and retinoid cycles have been implicated in disease etiology, as well as major structural components of photoreceptors (for extensive reviews of gene function see 26, 27; Tables 1–3). RP is the most common form of IRD, affecting approximately 1 in 3,000 people (4,5), the disease being characterized by progressive loss of rod photoreceptor function and their demise, followed by the death of the cone photoreceptors, initial symptoms of nyctalopia (night blindness) being followed usually by extensive loss of daytime vision as cones degenerate. Mutations within around 70 disease-causing genes have so far been implicated in RP (www.sph.uth.tmc.edu/retnet/; date last accessed April 25, 2017), while other genes still remain to be identified, indicating that this condition may, perhaps, be better regarded as a cluster of related conditions with similar clinical presentations.

## Next-Generation Sequencing of IRD Populations

**Figure 1:**
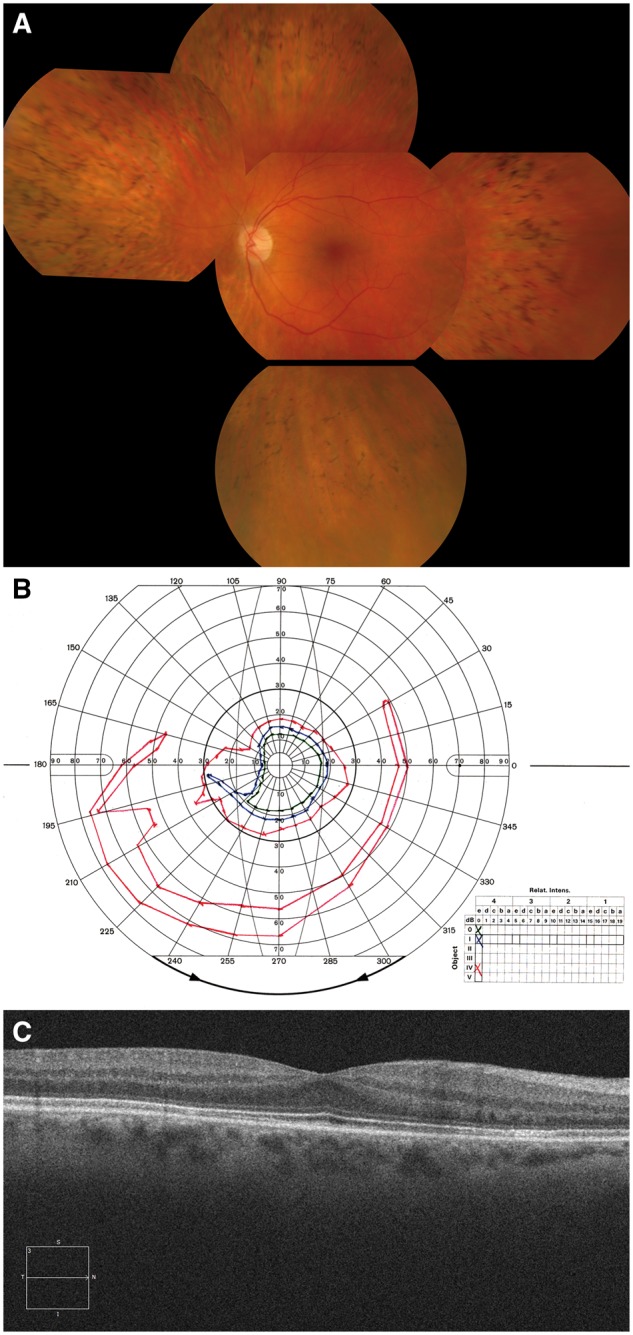
SLC24A1 causative of an autosomal recessive retinal degeneration. **Panel A**: Fundus photograph of the right eye of the proband with an SLC24A1 mutation indicating mild optic disc pallor, moderate arteriolar attenuation and quite extensive thinning of the retinal pigment epithelium in the retinal mid-periphery with abundant bone spicule intra-retinal pigment deposits, typically seen in patients with Retinitis Pigmentosa. **Panel B**: Goldmann kinetic perimetry of the left eye. Marked concentric constriction is evident, even to the large IV4e target (red), with preservation of an inferior island of field. This pattern of visual field loss is very typical of that seen in Retinitis Pigmentosa. **Panel C**: Optical Coherence Tomography (OCT) image of the left macula showing preservation of retinal structures, consistent with the normal appearance of the macula in Panel A and the patient’s good best-corrected visual acuity of 6/6.

Future exome and whole genome sequencing, in conjunction with emerging methods to define the function of candidate mutations in regulatory and intronic regions and to better characterize copy number variations (CNVs), all of which have been implicated as causative of some forms of IRD, will undoubtedly progress our understanding of the molecular genetics of IRDs to a new level. The situation for IRDs is further complicated by the diversity of clinical presentations that can be caused by mutations even within a single IRD gene, as well as overlapping clinical phenotypes which may be the result of mutations in entirely distinct genes, making it impossible for a diagnosis to be given in the majority of cases on the basis of disease phenotype alone (17,28–30). By way of example, in a recent NGS study of an Irish IRD patient cohort, new disease phenotypes were associated with the GNAT1 and SLC24A1 genes (28). In both cases a severe homozygous mutation in a known congenital stationary night blindness (CSNB) gene caused a late-onset form of RP involving photoreceptor loss (Fig. 1). Furthermore IRD phenotypes can be influenced by modifier loci (31–33). Given the limitations associated with the clinical diagnosis of specific forms of IRD, it is no surprise that the majority of patients with IRDs do not know the mutation(s) responsible for their condition. Recent advances in the development of gene-specific therapies for IRDs has made this issue all the more pressing, since this offers, for the first time, the prospect of modulating, halting or reversing the degeneration associated with some of these conditions (34), however, such therapies cannot, obviously, be administered without a precise knowledge of the underlying mutation to be treated.

High throughput NGS technologies offer an opportunity to rapidly characterize causative mutations in the growing number of IRD genes that have now been identified. These technologies have greatly accelerated DNA sequencing by providing a means of simultaneously sequencing many small fragments of DNA from different regions of the genome in a single reaction, and moreover by barcoding patient DNA to enable sequencing of samples from multiple patients in parallel. The short sequence reads from such fragments are then reconstructed by comparing the sequence to a reference genome; hg38 being currently the reference genome of choice for many such studies (hg38; GCA_000001405.15). NGS can be undertaken on the whole genome, or limited to expressed (coding) sequences (whole exome), or targeted to particular regions of the genome (employing target capture panel NGS); where genes of interest are, in essence, captured and sequenced. This form of sequencing has advanced at an exponential pace, with the cost per megabase of sequence halving regularly (35) and set to reduce further with the introduction of the new NovaSeq series from Illumina, among other innovations. IRDs have an ideal profile for NGS studies (14,18,36), representing a group of Mendelian conditions where many (to date approx. 260 genes), but not all of the causative genes, have been characterized. Given this scenario, NGS studies enable identification of known, or novel mutations in known genes and the stratification of IRD patient cohorts into those whose genetic pathogenesis has been resolved, and those for whom new genes, or variants in regulatory sequences, splice variants or CNVs associated with disease genes, may be causative of disease and therefore additional analyses deploying whole exome or whole genome sequencing would be appropriate.

**Figure 2:**
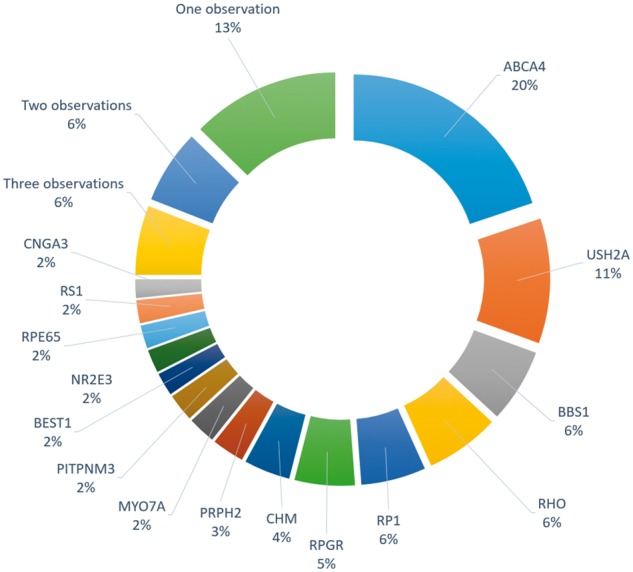
Frequencies with which different genes were found to harbour disease-causing mutations in the Target 5000 study in Ireland (Carrigan *et al*. 2016), counted as number of independent pedigrees. Genes that were observed less than four times, could not be cleanly visually represented, and so are listed below. Three observations: ADGRV1, CERKL, CRX, EYS, KLHL7 Two observations: CNGB3, CRB1, NRL, PROM1, PRPF8, RP2, SNRNP200, TRPM1 One observation: ABCC6, ABHD12, AIPL1, BBS10, BBS4, BBS9, C2ORF71, CDH23, CLRN1, CNGA1, ELOVL4, EMC1, FZD4, GNAT1, GUCY2D, HK1, HMCN1, IFT140, IMPDH1, IQCB1, LCA5, MFN2, MFRP, NMNAT1, NYX, OAT, PDE6A, PRPF31, RDH5, SDCCAG8, SLC24A1.

A variety of NGS studies, many employing a target panel-based NGS approach focused on sequencing the exons of known IRD genes, have been undertaken (10–25; Table 1). These studies support the view that IRDs represent an exemplar group of disorders for the application of panel-based NGS or WES as effective tools for detection of causative mutations. From such studies, some common patterns in findings have been observed, as have some unique findings, which at present, remain specific to individual studies. Of note, using target-capture based NGS, approximately 50–60% of IRD patients were found to carry disease causing or likely disease causing mutations in many studies. Given additional family studies indicating segregation of the disease, and replication of the findings in a certified diagnostic laboratory, these patients would be deemed to be categorised as ‘resolved’ in terms of the genetic etiology of the retinal pathology. Among recent examples of capture-panel NGS for IRDs, is a study of 537 IRD patients in which the variants identified were deemed to account, or likely account for the disease in 51% of cases and a recent study of Italian IRD patients, in which 59% of cases were resolved, similar to the identification levels obtained in many other NGS studies (17,19; Tables 1–3). Detection rates were significantly affected by the range of conditions under study, the number of genes included in the capture panel, as well as whether the rate was corrected based on previous screening of the same population, making precise comparisons of detection rates between studies challenging. Nonetheless, there is a consistent finding that between 25-50% of cases were not solved by targeted sequencing or WES. It is of interest that studies employing WES, rather than capture-panel NGS, have resulted in the identification of the underlying genetic defect in similar levels of IRD patients ∼60% (22), although in a recent study of Spanish IRD patients, WES resulted in successful identification of pathogenic variants in approximately 70% of patients (24). Levels of mutation detection achieved for IRDs that are less genetically heterogeneous, such as choroideremia, involving solely the CHM gene, and Stargardt disease, predominantly caused by ABCA4 mutations, were typically significantly higher (18; Fig. 2). However, even within such comparatively homogenous forms of IRD, the level of intragenic mutational heterogeneity present is still being characterised. For example, in a recent study in which 148 pathogenic or likely pathogenic mutations in the ABCA4 gene were identified, about a third of these (n = 48) represented new Stargardt associated disease alleles (37).

## Deciphering the Genetic Pathogenesis of Unresolved IRDS

It is evident from this body of research that currently the genetic pathogenesis of IRDs remains unresolved in ∼40% of cases (10–25; Tables 1–3). IRD mutation detection may remain elusive for a variety of reasons, possibly due to inadequate sequence coverage, inappropriate filtering of data (and loss of the pathogenic variant), variants in intronic sequences which affect splicing (such sequences do not form part of most capture panels), structural variants such as inversions, duplications and so on which can be difficult to resolve with NGS, regulatory variants or new, as yet uncharacterized, IRD genes and which therefore were not included in capture panels, among many other causes. However from some NGS studies there are indicators that support the hypothesis that, within the known IRD genes included in many target capture panels, additional significant levels of pathological genetic variants are present but as yet remain undetected. For example, it has been found that there can be a preponderance of patients with one causative mutation in a recessive IRD gene; such over-representation of heterozygotes compared to a control population has been observed in a number of NGS studies, including our own study of the Irish IRD population, for example, for the ABCA4 gene causative of Stargardt disease. The majority of clinically defined Irish Stargardt cases who did not have two ABCA4 mutations in the coding sequence, had a single ABCA4 mutation and this was highly significantly enriched compared to the frequency of ABCA4 mutant heterozygotes in a control Irish population (Carrigan M, unpublished data). Recent studies have focused on revealing the genetic variants underlying such observations. Various tools have been developed to detect copy number variations (CNVs) in NGS datasets and have been employed to successfully detect CNVs in IRD patient cohorts (38–40). Indeed, CNVs were identified in a recent WES study in approx. 10% of the 60 IRD patients assessed by exon coverage data analysis and confirmed by PCR (39). Alternative methods for CNV detection, that previously have been employed extensively in diagnostic laboratories for disorders other than IRDs, include quantitative PCR (qPCR), multiplex ligation-dependent probe amplification (MLPA) and comparative genomic hybridization. Using the latter, Van Cauwenbergh and colleagues developed a custom microarray (arrEYE) with coding and noncoding sequence from 166 known and candidate IRD genes and 196 noncoding RNAs for CNV detection in IRD patients (40); the CNV detection rate obtained with arrEYE from a first study of 57 IRD patients was 3.5%. In a recent study, 18% of unresolved cases (n = 28) were resolved by CNV mapping (38). It is clear that CNVs may represent a significant contributor to the unresolved cases of IRDs and that methods of CNV analysis, both computational and experimental, should, where possible be included in future IRD studies, which has not always been the case thus far.

In addition to CNVs, a proportion of the remaining unresolved IRD cases may be caused by variants that affect RNA splicing and thereby contribute to disease. Mutations that may have an impact on pre-mRNA splicing can be predicted using various *in silico* tools such as Human Splicing Finder (www.umd.be/HSF/; date last accessed April 25, 2017) among others, and can be confirmed by transcript analysis. RNA sequencing technologies are greatly enabling high throughput transcriptomics and undoubtedly will be applied to a greater extent to explore splice variants in IRDs in the future. A transcriptomic approach to assess the effects of IRD mutations on splicing may in principle be readily adopted for ubiquitously expressed IRD genes, and for tissue specific IRD genes, using iPS cells differentiated into appropriate cell lineages as per the example above. One approach to identify intronic variants with effects on RNA splicing in IRD patients involves deep intronic sequencing. For example, in a recent study of the USH2A gene, the most frequent cause of Usher syndrome type II (USH2) involving RP and sensorineural hearing loss, an analysis of the whole 800kb of the USH2A gene was prompted by the identification of patients with an USH2 phenotype and a single exonic mutation in the USH2A gene. Deep sequencing of intronic sequences enabled the identification of candidate splice mutations and subsequent functional validation of these mutations using reporter minigene assays leading to the resolution of 3 out of 5 USH2 patients with a single exonic mutation (41). In another study, the functional effect of a commonly observed mutation in the ABCA4 gene (the c.5461-10T→C variant) causative of Stargardt disease was analysed using patient-derived fibroblasts reprogrammed into induced pluripotent stem (iPS) cells and then differentiated into photoreceptor progenitor cells. This ABCA4 variant was found to induce skipping of exon 39 or exon 39 and 40 in the mature transcript again using a minigene assay (42). Recently homozygosity mapping and whole genome sequencing revealed a variant deep in intron 18 of the PROM1 gene causative of a recessive cone-rod dystrophy, resulting in the inclusion of a pseudo-exon in the mutant transcript, which was functionally validated again using a minigene assay (43). Detection of pathogenic IRD intronic variants will elude many of the current capture panel NGS strategies for IRDs which are focused solely on exons and hence the extension of screening studies in the future to include intronic sequences will aid in elucidating what proportion of IRD cases involve aberrant splicing.

In addition to CNVs and splice variants as a source of as yet ‘unresolved’ pathogenic IRD mutations, variants in regulatory sequences, such as, promoter sequences or miRNAs, and their associated target sites, may also be implicated in some forms of IRD. Indeed a mutation in the seed region of microRNA-204 has been found to segregate in a family with autosomal dominantly inherited retinal dystrophy and bilateral coloboma (44), a 5’UTR sequence in the NMNAT1 gene has been implicated in a form of LCA (45) and recently a single base mutation in the promoter region of the CHM gene has been implicated as causative of choroideremia (46), all highlighting the potential role of regulatory mutations in some forms of IRD. The implementation of more extensive NGS strategies in the future will aid in characterising such IRD regulatory mutations.

In conclusion, while significant advances in high throughput genomic and transcriptomic NGS has greatly facilitated an elucidation of much of the genetic architecture of IRD patient cohorts, substantial levels of unresolved IRD cases still remain. Recently it has become evident that a significant proportion of these will be accounted for by CNVs, splicing defects and regulatory variants. It still remains to be established within these unresolved cases, what proportion will be caused by new, as yet uncharacterised, retinal disease genes.

## A Diagnostic Imperative Driven by Developments in Gene Therapy

There is an obvious rationale for pursuing such studies to their conclusion,in that gene therapies for a growing number of ocular disorders are now in clinical trial (47–51). To date, approximately 300 IRD patients have been treated with ocular gene therapies (www.clinicaltrials.gov). In some of these trials, a continued retinal degeneration has been observed (52) and the parameters determining this still need to be elucidated. It is of note however, that an adeno-associated virus (AAV)-RPE65 therapy (voretigene neparvovec) has successfully progressed through to Phase III clinical trial (CHOP/Spark Therapeutics; www.sparktx.com), as has an AAV-ND4 therapy for Leber hereditary optic neuropathy (LHON) (51). Data from many ocular gene therapy trials support the view that intraocular delivery of AAV is well tolerated in the human eye and hence represents a safe platform for gene delivery (48–51). A large number of gene therapies employing AAV vectors are also at the stage of preclinical evaluation in animal IRD models, in which benefit has been demonstrated (51,53–57). Thus far many gene therapies have been directed towards recessive forms of IRD, although strategies such as suppression and replacement or genome editing are being considered to address the 30% of IRDs that are dominantly inherited (53,58). The efficacy of the therapeutic approaches will be determined in part by features specific to individual IRDs. For example, the target cell type within the retina, whether photoreceptors, RPE cells or retinal ganglion cells (51,53–57), among others, will influence vector choice and route of administration. Strategies such as directed evolution are also expediting the generation of AAV serotypes with a predilection for specific retinal cell types (59,60). Additionally, the severity of disease and whether retention of a relatively intact target cell population is maintained over years, thereby providing a significant timeframe for therapeutic intervention, will also greatly influence therapeutic efficacy. For severe degenerative IRDs, where few or no photoreceptors remain, optogenetic therapies may provide an alternative option by enabling remaining cells to become photosensitive (61,62). While AAV has shown substantial promise for a number of ocular indications, preclinical and clinical studies have also been undertaken with a variety of other non-viral and viral vectors, for example, nanoparticles and lentiviral vectors amongst others (63,64). Moreover, methodologies for systemic delivery of potentially therapeutic compounds into the retina by modulation of permeability at the inner blood-retina barrier have barrier have been extensively tested in animal systems and have employed AAV vectors (65). Given the rapid pace at which therapies are being developed, it is imperative to establish the genetic architecture of IRDs in different national and ethnic groups, in principle thereby facilitating participation of patients in future human clinical trials or access in the future to marketed gene therapies. In parallel with genotyping of IRDs, it is vital that patients are clinically profiled over time, as this will greatly augment our understanding of the natural histories of these ocular disorders and genotype-phenotype correlations. Knowledge regarding the natural history of a disorder is of direct value to patients, and moreover, will in principle facilitate future patient participation in clinical trials and will aid in informing the choice of appropriate primary and secondary endpoints for clinical trial design. Therefore as such, identification of the disease-causing mutations is of immediate clinical relevance to the entire IRD patient population.

*Conflict of Interest statement.* NC, GJF, PH, PK, AP & SMW are shareholders in Spark Therapeutics. 

## Funding

Vision research at the Ocular Genetics Unit at TCD is supported by awards from Fighting Blindness Ireland (FB Irl), the Health Research Board of Ireland (HRA_POR-2013-376; HRA_POR-2015-1140), the Medical Research Charities Group (MRCG-2012-4; MRCG-2013-8; MRCG-2016-14), the Irish Research Council (IRC), Science Foundation Ireland (SFI 11/PI/1080) and the European Research Council (ERC-2012-AdG 322656-Oculus). Funding to pay the Open Access publication charges for this article was provided by Science Foundation Ireland.
